# The Pattern of Medicine Use in Ethiopia Using the WHO Core Drug Use Indicators

**DOI:** 10.1155/2021/7041926

**Published:** 2021-12-24

**Authors:** Solomon Ahmed Mohammed, Abebe Getie Faris

**Affiliations:** Department of Pharmacy, College of Health Science, Wollo University, Dessie, Ethiopia

## Abstract

**Introduction:**

Rational medicine use is an appropriate prescribing, dispensing, and patient use of medicines for the diagnosis, prevention, and treatment of diseases. It is affected by several factors. Irrational use of medicine is a widespread problem at all levels of care. This review is aimed at assessing the medicine use pattern in health facilities of Ethiopia using the medicine use pattern developed by WHO/INRUD.

**Methods:**

Relevant literature was searched from Google Scholar, PubMed, Hinari, Web of Science, and Scopus using inclusion and exclusion criteria. A systematic review was used to summarize the medicine use pattern in health facilities of Ethiopia, and that WHO core drug use indicators were employed.

**Result:**

From 188 searched studies, 30 literatures were reviewed. The average number of drugs per encounter was 2.11. The percentage of encounters with antibiotics and injection was 57.16% and 22.39%, respectively. The percentage of drugs prescribed by generic name and from an essential drug list was 91.56% and 90.19%, respectively. On average, patients spent 5.14 minutes for consultation and 106.52 seconds for dispensing. From prescribed drugs, 67.79% were dispensed, while only 32.25% were labeled adequately. The availability of key essential medicines was 64.87%. The index of rational drug use value was 7.26. Moreover, the index of rational drug prescribing, index of rational patient-care drug use, and index of rational facility-specific drug use were 3.74, 2.51, and 1.01, respectively.

**Conclusion:**

Ethiopian health facilities were faced with antibiotic overprescribing, short consultation, and dispensing times, poor labeling of medicines, poor availability of key drugs, and nonadherence to the essential drug list. Routine, multidisciplinary awareness creation, and regulation should be implemented to promote rational medicine use at a national level.

## 1. Introduction

Medicines are one of the most common therapeutic interventions and a crucial component of medical care for any healthcare system [1]. Hence, developing countries spent 20-50% of the budget on medicine, and medicine expenditure estimated 60–80% of their populations [2]. The appropriate use of medicine is essential for optimizing the health of individual patients and the population of any nation [3].

Rational drug use (RDU) includes appropriate prescribing, dispensing, and patient use of medicines for the diagnosis, prevention, and treatment of diseases [4, 5]. Rational prescription practices have medical, social, and economic implications. To promote RDU, the patient should receive medicines appropriate to their health care conditions, at optimum doses and sufficient time, as well as at the cost that the individual and the community [4].

Rational drug use is affected by several factors such as economy, funds, manpower, culture, attitude and beliefs, knowledge gap, loose policy on medicines, load on health professionals, and inappropriate promotion of medicines [6]. These factors made irrational use of medicines a widespread problem at all levels of care and results in increased mortality, morbidity, reduction in quality of medicine therapy, increase cost of therapy, adverse drug reactions, enhanced microbial resistance, poor patient outcomes, and wastage of scarce resources [7]. The problem worsens in developing countries due to inadequate funds for medicine procurement, inadequate training of prescribers, attitudes of prescribers, and beliefs of patients [4].

Inappropriate use of medicines characterized by polypharmacy, indiscriminate and frequent use of injections and antibiotics, and use of brand names in prescribing and prescribing medications not by from essential drug list (EDL) [8]. Globally, more than 50% of all medicines are prescribed, dispensed, or sold inappropriately, while 50% of patients fail to take them correctly [9]. Even though the problem is common in both developed and developing countries, the magnitude is higher in developing countries [10].

Following the Declaration of Primary Health Care (1978) and its call for Health for All (2000), the Ethiopian Government welcomed and started implementation. However, the national health policy based on the declaration alone was largely unsuccessful at first due to lack of clarity in specific element of policies and strategies as a result of poor and inadequate dissemination of information and limited awareness [11]. Currently, the Ethiopian healthcare system is structured in a three tier system: primary, secondary, and tertiary level of care. The primary level of care includes primary hospital, health center, and health post. The primary health care unit comprises five satellite health posts and a referral health to administered and facilitate the first level of care [12].

Preventive, promotive, and basic curative services were included under the primary health care service. Health Extension Program was introduced in 2002 to enhance primary health care services [13]. The government of Ethiopia allocated United Stated $ 1.6 billion to health care in 2015. Of total health expenditure, 14.69% goes to finance primary health care [14]. Health care provision depends on efficiently combining financial resources, human resources and supplies, and delivering services in a timely fashion and with equitable spatial distribution throughout a country [15].

However, the program is facing with lack of medical equipment and medicine, deficient supply chain management and quality assurance, low ratio of health professionals to population, and lack of quality and competency among health professionals [16]. Despite the governance-developed indicators in the aggregate, indicators for specific sectors, like health, are often not readily available. Consequently, it is necessary to look for standard indicators that reflect the quality of health provision [17].

Despite the process of diagnosis and pharmaceutical care is complex, the uses of medicines are necessary to be evaluated regularly to define the improvement of medical utilization. In 1985, the World Health Organization (WHO) convened a conference in Nairobi and developed an essential tool to investigate medicine use in health facilities [18]. The WHO developed core drug use indicators that are used as measurement tools for identifying and analyzing and promoting rational use of medicines in developing countries. This WHO core drug use indicators are prescribing, patient care, and health facility indicators [3].

Prescribing indicators is one of the core medicine use indicators that include the average number of drugs per encounter, the percentage of drugs prescribed with generic names, the percentage of prescriptions with antibiotics, the percentage of prescriptions by injection, and the percentage of prescribed drugs from the list of essential medicine or formularies. The patient service or care indicator is also the core drug use indicator consisted of the average consultation time, the average time of preparation of the medicine, the percentage of the right medicine given, the percentage of the drug that was adequately labeled, and the patient knowledge of the appropriate dose. Indicators of health facilities are the availability of a list of essential medicine or formulary and the availability of important medicines [4, 9].

A systematic review and meta-analysis on medicine use among outpatients at healthcare facilities in Ethiopia showed that prescription of antibiotics and medicines was higher than the reference values and increasing over the years [19]. Another systematic review and meta-analysis also reported that all of the prescribing indicators were not consistent with the standard values [20]. The practice of rational medicine use varied across the region of the country and deviation was also reported from the standard recommended by WHO [21, 22].

Despite a few reviews were done before, none of the previously done articles addressed all the three types of WHO core drug use indicators. Moreover, for a comprehensive appraisal of medical care, this review used the indices for further understanding of rational medicine use practice. Thus, this systematic review identified all empirical evidence to answer the practice of rational drug medicine in Ethiopia. This review was aimed at assessing the medicine use pattern in health facilities in a reproducible manner using the medicine use pattern developed by the WHO/international network for rational use of drugs (INRUD).

## 2. Materials and Methods

### 2.1. Search Strategy

**Figure 1:**
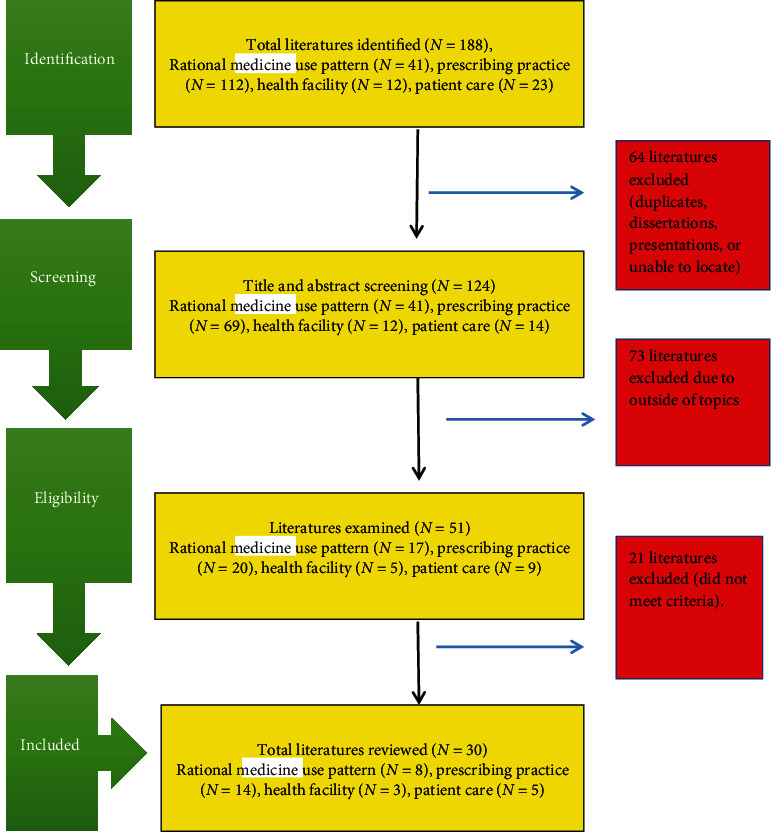
Data searching process.

A systematic literature search was conducted in Google Scholar, PubMed, Hinari, Web of Science, and Scopus electronic databases for articles published between January 2000 and May 2020. The authors set 2000 as a lower limit to include more empiric findings. This would help to have a better understanding of rational medicine use practice and the progress of medicine use as well. Some studies were also identified through a manual Google search and the reference lists of retrieved articles. The entire searches were done from May 13-14/2021 using keywords “rational medicine use pattern,” “WHO indicator,” “prescribing practice,” “health facility,” “patient care,” and in combination. For the identification of articles to be included in this review, Boolean operators (AND, OR) and truncation were used properly. From these databases, a total of 188 literatures were extrapolated. The complete data searching process from all databases and complete list of the search strategies used in each database was presented in supplementary information 1 and 2, respectively. After the exclusion of redundant and irrelevant literature, a total of 30 separate published empirical articles in peer-reviewed journals and one gray literature were reviewed. From this, 8 articles assessed the three types of medicine use pattern, 14 articles assessed prescribing practice, 3 articles assessed health facility, and 5 articles assessed patient care indicators. The searching process is displayed in Figure 1.

### 2.2. Article Selection

The studies that assess medicine use patterns using WHO/INRUID core drug use indicators were included in the review. Studies that were written in English, open access in portable document formats and all study designs were included, while those studies published only as dissertations, abstracts, editorials, or clinical opinion, and published before 2000 were excluded. Dissertations were not included as they are not peer-reviewed and therefore may be less scientifically rigorous than those that are peer-reviewed and published.

### 2.3. Data Abstraction and Analysis

Data were extracted by two reviewers independently. The author, study area, year, study design, sample size, sampling technique, and WHO/INRUID core medicine use indicators used for evaluating the rational medicine use were extracted from each study using data abstraction form. The authors summarized the findings in narrative summary. Quantitative synthesis was done to calculate the Index of Rational Drug Prescribing (IRDP).

The IRDP was calculated for all health centers by adding the index values of all prescribing indicators. Then, the Index of Rational Patient-Care Drug Use (IRPCDU) and the Index of Rational Facility- Specific Drug Use (IRFSDU) were calculated. Finally, the Index of Rational Drug Use (IRDU) was calculated for all health centers by adding up the total of IRDP, IRPCDU, and IRFSDU. A total Index of Rational Drug Use (IRDU) was calculated by adding up the total of IRDP, IRPCDU, and IRFSDU. Ranking of health centers was done based on these indices. The health centers with higher IRDU value were considered the best performing in terms of rational medicine use and were given the first rank. The optimal index for all indicators was 1. The closer to 1, the more rational medicine use indicators and vice versa [23]. The final summary indices would help to assess whether rational medicine was practiced or not.

### 2.4. Assessment of Methodological Quality

Methodological validity was checked before the inclusion of selected articles and during the review by undertaking critical appraisal. The risk of bias for individual studies was assessed by using Cochrane Handbook for Systematic Reviews of Interventions: Assessing Risk of Bias in Included Studies [24].

## 3. Results

### 3.1. Result of Methodological Quality Assessment

There are several ways to rate validity. Since few explicit criteria were used to assess validity, we summarize the overall assessment of how valid the result of the study is. Thus, three categories such as “low risk bias” if plausible bias unlikely to seriously alter the result, “moderate risk bias” if plausible bias that arise some doubt about the result, and “high risk bias” if plausible bias that seriously weakens confidence in the result. The criteria were random sequence generation (selection bias), blinding of participants and personnel (performance bias), blinding of outcome assessment (detection bias), blinding of outcome assessment (detection bias), incomplete outcome data addressed (attrition bias), incomplete outcome data addressed (attrition bias), and selective reporting (reporting bias). The validity was set as follows; if all of the criteria were met “low risk bias,” if one or more criteria partly met “moderate risk bias,” and if one or more criteria partly not met “high risk bias.” The result of validity quality assessment was presented in supplementary information 3. The process of validity assessment was executed by two independent reviewers. Each reviewer appraised the full text of each study independently. Any discrepancies between the two reviewers was resolved through discussion.

### 3.2. Summary Results of Included Articles

All included studies were cross-sectional. Twenty-five studies assessed the medicine use at the hospital, three assessed at the health center, and two assessed at both setups. The number of surveyed health institutions was ranged from 1 to 8. Twenty studies used a retrospective cross-sectional design, while three studies used retrospective and prospective cross-sectional design. A detailed description of the characteristics of individual studies is displayed in Table 1 (Table 1).

### 3.3. WHO/INRUID Prescribing Indicators

On average, 2.11 medicines were prescribed. The total number of drugs prescribed by generic name was 91.56%. An antibiotic was prescribed in 57.16%, and an injection was prescribed in 22.39% prescription. Twenty-seven studies that evaluated the prescription pattern based on the WHO/INRUID prescribing indicator were reviewed. Of them, four studies (16%) meet WHO recommendations on an average number of drugs per encounter, while one study meets the WHO recommendation on the percentage of encounters with antibiotics. The WHO recommends all medicines should be prescribed by generic name and from an essential list of medicine or formulary. Two of the studies (8%) reported prescribed drugs by generic name and five of the reviewed studies (20%) prescribed drugs from the essential medicine list. Four of the studies (16%) were in line with the WHO recommendation on the percentage of encounters with injection (Table 2).

### 3.4. WHO/INRUID Patient Care Indicators

The average consultation and dispensing time were 5.14 minutes and 106.52 seconds, respectively. Of total prescribed medicines, 67.97% were dispensed; off this 33.5% were adequately labeled. Thirteen studies that evaluated the rational medicine use pattern based on WHO/INRUID patient care indicators were reviewed. Only one and three studies were consistent with WHO/INRUID recommended average consultation and dispensing time, respectively. None of the studies meets the WHO/INRUID rational medicine use recommendation on the percentage of medicines dispensed and percentage of medicines adequately labeled (Table 3).

### 3.5. WHO Health Facility Indicators

The percentage availability of the WHO model list of key essential medicines was ranged from 50-96.7. Four studies revealed the availability of standard treatment guidelines (STG), while the availability of key essential medicines was below the standard (Table 4).

The analysis of the index revealed that the Index of Rational Drug Use (IRDU) value was 7.26. Among specific indices, Index of Rational Drug Prescribing (IRDP) was 3.74, Index of Rational Patient-Care Drug Use (IRPCDU) was 2.51, and Index of Rational Facility- Specific Drug Use (RFSDU) was 1.01 (Table 5).

## 4. Discussion

Rational medicine use requires that patients receive medications to coincide with their clinical needs [25, 26]. It reduces the occurrence of undesired toxicity and adverse events and maximizes the benefits that can be derived from the optimal use of scares health care costs [27]. Overuse of medicines, inappropriate use of antibiotics, and overuse of injectables were the common types of irrational medicine use that could lead to poor treatment outcomes, drug-drug interactions, and high economic burden, and the worst-case loss of the patient's life [28]. Inappropriate prescribing of medicine may have a negative impact because some medicines prescribed are known to be toxic to specific populations and may lead to under-use of effective medicines, especially antibiotics [29, 30].

Among twenty-seven reviewed studies done on the evaluation prescription pattern based on WHO/INRUID prescribing indicator in Ethiopia, only four studies meet WHO recommendation on an average number of drugs per encounter (1.6-1.8). This showed that more than two medicines were prescribed within the single prescription in Ethiopia. Polypharmacy was also reported in systematic reviews and meta-analyses done in Ethiopia [19, 31]. This misuse of available resources by loading the patients with unnecessary medicines or sending prescriptions to private retailers and thereby expose patients to expensive private sector spending [32]. Thus, health care providers should reduce polypharmacy since it increases drug interaction, adverse drug effects, and cost [30].

Only one study meets the WHO recommendation on the percentage of encounters with antibiotics (20-26.8); however, all of the studies showed that there was overuse of antibiotics in Ethiopia. This will increase bacterial resistance of antibiotics, nonadherence of the patient, increase the necessity to use more expensive antibiotics to treat the common and life treating condition, and result in poor outcomes for the patient. This might be due to the absence of strict rules and regulations in the country regarding antibiotic prescription [6].

This review revealed that only two of the reviewed studies showed complete medicines prescription by generic name. The percentage of generic drug use was ranged from 70.5% to 100% [21]. The lower level of prescription by generic name was also reported by other systematic reviews and meta-analyses [22, 31]. However, WHO recommends all medicines should be prescribed by generic. This low rate of prescribing medicines by generic name might be due to poor prescribers' awareness about advantages of generic prescription, poor countries' medication procurement policy which promotes procurement by generic, and absence of good discussion among health care providers in various professional sessions such as case presentation and medicine and therapeutic committee meeting [28]. Presence of more brand for specific generic medicine confuses the patient and health care provider and results in a medication error [30, 33]. There should be strict adherence to generic guidelines to reduce the cost of medical therapy and rationalize therapy. Moreover, the availability of standard treatment guidelines that can be used as an information and educational tool for health care professionals should be improved.

Four of the studies done on the prescribing practice meet the WHO recommendation on the percentage of encounters with injection. This finding was consistent with other systematic reviews and meta-analyses [22, 31]. However, some deviation from the standard value was also reported [19]. The overuse of injections leads to an economic load on the patient since they are very costly, and nonsterile injections enhance transmission of hepatitis and other blood-borne diseases. To improve the appropriate use of injections, the prescribers and dispensers should communicate in the choice of appropriate dosage form for the patients. Moreover, the presence of more, affordable, easier, and appropriate oral formulation routine use of injection should be discouraged [28].

Patent care indicators are used for assessing rational medicine use. In Ethiopia, twelve studies evaluated rational medicine use patterns based on WHO patient care indicators, and only one study [34] reported that the pharmacist consulted appropriately in comparison with WHO recommendation (ten minutes) [35]. An average dispensing time between 6.74 minutes and 0.7 minutes was reported by a systematic review [21]. This might be due to patient load and lack of knowledge. Good communication between health care providers and patient helps them to get enough information about their medications and enhance adherence [36].

Percentage of medicines dispensed and percentage of medicines adequately labeled did not meet the WHO rational drug use recommendation. The result was in line with other systematic reviews [21]. This might result medication error, medicine-related adverse event, and therapeutic failure. Poor labeling practice may be due to patient load, poor attitude from the dispenser, and lack of resources. Adequate labeling medicine is used to uniquely identify the content of the container and to ensure that the patient has clear and concise information about the use of medicine [37]. Educational, managerial, and regulatory strategies are highly recommended to reduce the degree of irrationality to off-set from medicine misuse [30].

The present study revealed that the overall IRDP was 3.74. The value was lower than the ideal value of 5. However, the IRDP was higher than Sierra Leone (2.6) [38], but lower than Pakistan (3.77 to 5) [39], Saudi Arabia (3.78 to 4.27) [40], and Egypt (3.92 to 4.88) [41]. The result of IRDP indicated relatively lower medicine use practices compared with other regions which urge that the prescribing practice should be improved more.

This review had the limitation. We restricted the time of publication for the identification of potentially eligible studies. The findings of this review will serve to stimulate further questioning and guide subsequent action. Moreover, it will help to improve the performance of health professionals' behavior and health facilities in promoting rational medicine use.

## 5. Conclusion

Ethiopian health facilities were faced antibiotics overprescribing, short consultation, and dispensing times, poor labeling of medicines, poor availability of key medicines, and nonadherence to essential medicine lists. The average number of drugs prescribed per encounter was slightly higher than the standard. Routine, multidisciplinary awareness creation and regulation should be implemented to promote rational medicine use at a national level.

## Figures and Tables

**Table 1:** Study characteristics of reviewed articles.

Sr no	Author	Study area	Number of surveyed health facilities	Type of health facilities	Type WHO indicator studied	Study design	Sampling technique	Sample size	Year (GC)
1	Fereja and Lenjesa [34]	West Oromia region	4	Hospital	Health facility	Descriptive cross-sectional	Systematic random sampling	160	2012-2013
2	Bekele and Tadesse [42]	Southern Ethiopia	1	Hospital	Prescribing	Retrospective cross-sectional	Systematic random sampling	1440	2016-2018
3	Warsame [43]	Eastern Ethiopia	1	Hospital	Prescribing	Retrospective cross-sectional	Systematic random sampling	600	2018-2019
4	Balcha [44]	Southwest Ethiopia	1	Hospital	Prescribing	A retrospective cross-sectional, quantitative	Simple random sampling	384	2016
5	Lenjisa and Fereja [45]	West Ethiopia	4	Hospital	Prescribing	Retrospective descriptive cross-sectional	Simple random sampling	2024	2013
6	Dessie et al. [20]	Northwest Ethiopia	2	Hospital	All	Retrospective cross-sectional	Systematic random sampling	770	2019
7	Yilma and Liben [46]	North Ethiopia	1	Hospital	Prescribing	Retrospective descriptive cross-sectional	Systematic random sampling	384	2016-2017
8	Mishore et al. [47]	Eastern Ethiopia	1	Hospital	Prescribing	Retrospective descriptive cross-sectional	Simple random sampling	344	2018
9	Desalegn [48]	Southern Ethiopia	1	Hospital	Prescribing	Retrospective cross-sectional	Systematic random sampling	1290	2007-2009
10	Angamo et al. [49]	Southwest Ethiopia	4	Health center	All	Prospective and retrospective cross-sectional	Systematic random sampling	140	2009
11	Mensa et al. [10]	Southern Ethiopia	2	Hospital	All	Retrospective and prospective cross-sectional	Systematic random sampling	1198	2013
12	Jabo et al. [50]	Addis Ababa	1	Health center	Prescribing	Retrospective cross-sectional	Stratified random sampling	11 and 40	2016
13	Wubetu et al. [51]	Northwest Ethiopia	2	Hospital	Prescribing	Retrospective cross-sectional	Systematic random sampling	362	2015-2016
14	Bilal et al. [52]	Eastern Ethiopia	8	Both	Patient care	Retrospective and prospective cross-sectional	Systematic random sampling	636and 708	2013-2014
15	Geresu et al. [33]	Northeast Ethiopia	1	Hospital	All	Retrospective and prospective cross-sectional	Systematic random sampling	361	2012
16	Summoro et al. [53]	Southern Ethiopia	4	Hospital	Prescribing	Retrospective cross-sectional	Systematic random sampling	1440	2014
17	Mamo and Alemu [54]	Northeast Ethiopia	1	Hospital	All	Retrospective cross-sectional	Systematic random sampling and convenience sampling method	500	2019
18	Sisay et al. [5]	Eastern Ethiopia	3	Hospital	Patient care prescribing	Retrospective cross-sectional	Systematic random sampling	1500	2014
19	Kasahun et al. [55]	North Ethiopia	1	Hospital	Prescribing	Facility-based cross-sectional	Systematic random sampling	600	2018-2019
20	Gashaw et al. [56]	Eastern Ethiopia	4	Hospital	Prescribing	Retrospective cross-sectional	Systematic random sampling	600	2016
21	Nigussie [57]	Northwest Ethiopia	8	Both	Health facility	Retrospective cross-sectional	Systematic random sampling	8	2014
22	Assen and Abrha [58]	Northeast Ethiopia	1	Hospital	Prescribing	Retrospective cross-sectional	Systematic random sampling	362	2013
23	Demeke et al. [28]	North Ethiopia	1	Hospital	Prescribing	Retrospective cross-sectional	Systematic random sampling	384	2014
24	Mosisa et al. [59]	West Ethiopia	1	Health center	Prescribing	Retrospective cross-sectional	Systematic random sampling	770	2014-2015
25	Mariam et al. [6]	Southern Ethiopia	1	Hospital	All	Retrospective and prospective cross-sectional	Systematic random sampling	384 and 30	2013
26	Gidebo et al. [53]	Southern Ethiopia	4	Hospital	A patient care, health facility	Retrospective cross-sectional	Systematic random sampling	384	2014
27	Dessie et al. [20]	Northeast Ethiopia	1	Hospital	Prescribing	Retrospective cross-sectional	Systematic random sampling	213	2010-2011
28	Gebramariam et al. [60]	West Shoa zone	7	Hospital	All	Retrospective and prospective cross-sectional	Systematic random sampling	21, 001, and 400	2017
29	Tefera et al. [61]	Eastern Ethiopia	1	Hospital	A patient care, prescribing	Retrospective cross-sectional	Systematic random sampling	600	2017-2018
30	Getahun et al. [62]	Northwest Ethiopia	1	Hospital	All	Retrospective and prospective cross-sectional	Systematic random sampling	1128	2019

**Table 2:** Medicine use pattern using WHO/INRUD prescribing indicators in Ethiopia (*N* = 27).

Author	The average number of drugs per encounter	Percentage of encounters with antibiotics	Percentage of encounters with an injection	Percentage of drugs prescribed by generic name	Percentage of drugs from essential drug list
Bekele and Tadesse [42]	1.8	58.47	6.53	85.33	97.43
Warsame [43]	1.98	60	2.5	89.5	98.99
Balcha [44]	2.1	32.05	1.35	93	100
Lenjisa and Fereja [45]	2.08	54.78	28.38	78.9	82.58
Dessie et al. [20]	1.93	75.1	6.49	97	—
Yilma and Liben [46]	1.96	58.6	42.2	90.4	86.3
Mishore et al. [47]	2.19	27.62	44.7	90.97	90.17
Desalegn [48]	1.9	58.1	38.1	98.7	96.6
Angamo et al. [49]	2.17	24.85	10.58	79.36	90.26
Mensa et al. [10]	1.86	54.44	10.01	100	100
Jabo et al. [50]	2.03	67.36	19.31	98.15	99.2
Wubetu et al. [51]	2.11	59.28	3.74	98.42	85.68
Bilal et al. [52]	2.2	82.5	11.2	97	92
Geresu et al. [33]	1.78	48.2	42	82.2	96.5
Summoro et al. [53]	2.09	66.48	37.65	96	95
Mamo and Alemu [54]	2.5	34.64	13.8	90.53	82.83
Sisay et al. [5]	2.34	57.2	10.87	90.89	—
Kasahun et al. [55]	1.78	49.2	4	95.63	99.5
Gashaw et al. [56]	2.17	61.38	26.49	89.4	89.24
Demeke et al. [28]	2.61	32	23.6	93.3	100
Mosisa et al. [59]	2.85	67	9	100	98.96
Mariam et al. [6]	2.3	70.6	20.3	96.8	88.7
Desse et al. [20]	2.4	71.36	48.36	77.69	98.24
Gebramariam and Ahmed [60]	1.74	48.9	12.6	96.7	100
Tefera et al. [61]	—	71.5	66	87.84	100
Getahun et al. [62]	1.88	37.5	20	91.4	91.4
Average	2.11	57.16	22.39	91.56	90.19
Standard	1.6-1.8	20-26.8	13.4-24.1	100	100

**Table 3:** Medicine use pattern using WHO/INRUD patient care indicators in Ethiopia (*N* = 13).

Author	Average consultation time (minute)	Average dispensing time (second)	Number of drugs prescribed	Percentage of medicines dispensed	Percentage of medicines adequately labeled
Fereja and Lenjesa [34]	18.16	393.6	422	2.16	25.03
Dessie et al. [20]	2.11	57	252	93.2	95.95
Angamo et al. [49]	6.15	76.8	217	83.39	70.08
Mensa et al. [10]	3.75	75.6	100	49.7	0
Bilal et al. [52]	5.6	162.6	—	86.54	64
Geresu et al. [33]	4.04	51.6	—	92.6	13.65
Mamo and Alemu [54]	1.57	47	362	82.6	22.7
Sisay et al. [5]	4.61	88.62	433	75.68	3.33
Mariam et al. [6]	5.5	73.2	78	89.7	25.71
Gidebo et al. [53]	4.8	110.4	1.9	1.4	48
Gebramariam and Ahmed [60]	5.12	76.8	—	73.21	0
Tefera et al. [61]	—	—	—	85.4	—
Getahun et al. [62]	0.25	65	—	—	18.5
Average	5.14	106.52	233.24	67.79	32.25
Standard	10	>180	—	100	100

**Table 4:** Medicine use pattern using WHO/INRUD health facility indicators in Ethiopia (*N* = 12).

Author	Availability of key essential drugs	Availability of standard treatment guidelines
Fereja and Lenjesa [34]	90.5	25
Dessie et al. [20]	—	0
Angamo et al. [49]	65	50
Mensa et al. [10]	73.31	—
Bilal et al. [52]	56.63	100
Geresu et al. [33]	65.7	100
Mamo and Alemu [54]	96.7	0
Sisay et al. [5]	50	0
Mariam et al. [6]	—	0
Gidebo et al. [53]	71.27	20
Gebramariam and Ahmed [60]	79.6	100
Getahun et al. [62]	74.56	100
Average	64.87	35.90
Standard	100	100

**Table 5:** Index of WHO/INRUD drug use indicators in Ethiopia.

Indicators	Index	Value
Prescribing indicators	Nonpolypharmacy index	0.85
	Generic name index	0.92
	Rational antibiotic index	0.47
	Injection safety index	0.60
	EDL index	0.90
	IRDP	3.74
Patient-care indicators	Consultation time index	0.51
	Dispensing time index	1
	Dispensed drugs index	0.68
	Labeled drugs index	0.32
	IRPCDU	2.51
Facility-specific indicators	Index of EDL	0.65
	Index of key drugs in stock	0.36
	IRFSDU	1.01
Grand total	IRDU	7.26

## Data Availability

The datasets are available from the corresponding author upon reasonable request.

## Supplementary Materials

Supplementary 1 Supplementary information 1: complete data searching process.

Supplementary 2 Supplementary information 2: complete list of the search strategies used in each database.

Supplementary 3 Supplementary information 3: result of methodological quality assessment.
