# Supplementary material for: The Pattern of Medicine Use in Ethiopia Using the WHO Core Drug Use Indicators
Source: Biomed Res Int. 2021 Dec 24;2021:7041926. doi: 10.1155/2021/7041926 (PMC8720245; doi:10.1155/2021/7041926)
Supplement: Supplementary 1 — Supplementary information 1: complete data searching process. [file 7041926.f1.docx]

Total literatures identified (N=188),

Google Scholar (N=47), PubMed (N=62), Hinari (N=29), Web of Science (N=26), and Scopus (N=24)

Identification

64 literatures excluded (duplicates, dissertations, presentations, or unable to locate)

Title and abstract screening (N=124)

Google Scholar (N=30), PubMed (N=34), Hinari (N=22), Web of Science (N=20), and Scopus (N=18)

**Screening**

73 literatures excluded due to outside of topics

**Eligibility**

Literatures examined (N=51)

Google Scholar (N=15), PubMed (N=13), Hinari (N=9), Web of Science (N=8), and Scopus (N=6)

21 literatures excluded (did not meet criteria).

Total literatures reviewed (N=30)

Google Scholar (N=9), PubMed (N=9), Hinari (N=5), Web of Science (N=4), and Scopus (N=3)

**Included**

Supplementary information 1: Complete data searching process
