# Supplementary material for: The Pattern of Medicine Use in Ethiopia Using the WHO Core Drug Use Indicators
Source: Biomed Res Int. 2021 Dec 24;2021:7041926. doi: 10.1155/2021/7041926 (PMC8720245; doi:10.1155/2021/7041926)
Supplement: Supplementary 2 — Supplementary information 2: complete list of the search strategies used in each database. [file 7041926.f2.docx]

Supplementary information 2: Complete list of the search strategies used in each database

| Database | Search strategy |
| --- | --- |
| Google Scholar | Rational medicine use pattern OR rational drug use pattern OR rational medicine use OR rational drug use AND ( “WHO indicator” OR “prescribing practice” OR “prescribing indicator” OR “health facility” OR “health facilities” OR “patient care” OR “patient care indicator”) |
| PubMed | Search (WHO indicator [Mesh] OR prescribing practice [Mesh] OR prescribing indicator [Mesh] OR health facility [Mesh] OR health facilities [Mesh]OR patient care [Mesh]OR patient care indicator) AND (rational medicine use pattern[Mesh] OR rational drug use pattern[Mesh] OR rational medicine use[Mesh]OR rational drug use[Mesh]) AND (Ethiopia OR Ethiopian) AND (‘2000/01/01’[Date-Publication]:’2020/04/13’[Date-Publication] AND (English[lang]) |
| Hinari | (WHO indicator [Mesh] OR prescribing practice [Mesh] OR prescribing indicator [Mesh] OR health facility [Mesh] OR health facilities [Mesh]OR patient care [Mesh]OR patient care indicator) AND (rational medicine use pattern[Mesh] OR rational drug use pattern[Mesh] OR rational medicine use[Mesh]OR rational drug use[Mesh]) AND (Ethiopia OR Ethiopian) AND (‘2000/01/01’[Date-Publication]:’2020/04/13’[Date-Publication] AND (English[lang]) |
| Web of Science | #1 [(WHO indicator*) OR (prescribing practice*) OR (prescribing indicator*) OR (health facility*) OR (health facilities*) or (patient care*) OR (patient care indicator*) AND (rational medicine use pattern*) OR (rational drug use pattern*) OR (rational medicine use*) OR (rational drug use*)]  #2 Research area = Pharmacy and pharmacology  #3 WOS category = Pharmacy and pharmacology  Search #1 and [#2 or #3] |
| Scopus | ((WHO indicator OR prescribing practice OR prescribing indicator OR health facility OR health facilities OR patient care OR patient care indicator) AND (rational medicine use pattern OR rational drug use pattern OR rational medicine use OR rational drug use)) |
