# Supplementary material for: The Pattern of Medicine Use in Ethiopia Using the WHO Core Drug Use Indicators
Source: Biomed Res Int. 2021 Dec 24;2021:7041926. doi: 10.1155/2021/7041926 (PMC8720245; doi:10.1155/2021/7041926)
Supplement: Supplementary 3 — Supplementary information 3: result of methodological quality assessment. [file 7041926.f3.docx]

Supplementary information 3: Result of methodological quality assessment

| Author | Random sequence generation (selection bias | Allocation concealment (selection bias) | Blinding of participants and personnel (performance bias) | Blinding of outcome assessment (detection bias) | Blinding of outcome assessment (detection bias) | Incomplete outcome data addressed (attrition bias) | Incomplete outcome data addressed (attrition bias) | Selective reporting (reporting bias) |
| --- | --- | --- | --- | --- | --- | --- | --- | --- |
| Fereja and Lenjesa | Low | Low | Low | Low | Low | Moderate | Moderate | Low |
| Bekele et al | Low | Low | Low | Low | Low | Low | Low | Low |
| Warsame MS | Low | Moderate | Low | Low | Low | Low | Moderate | High |
| Ayele et al | Low | Low | Low | Low | Low | Low | Low | Low |
| Lenjisa and Fereja | Low | Low | Low | Low | Low | Low | Moderate | Moderate |
| Dessie et al | Low | Low | Low | Low | Low | Low | Low | Low |
| Yilma et al | Low | Low | Low | Moderate | Moderate | Moderate | Low | High |
| Mishore et al | Low | Low | Low | Low | Low | Low | Low | Low |
| Desalegn AA | Low | Low | Low | Low | Low | Low | Low | Low |
| Angamo et al | Low | Low | Low | Low | Low | Low | Low | Low |
| Mensa et al | Low | Moderate | Moderate | Low | Low | Low | Moderate | Moderate |
| Jabo SA et al | Low | Low | Low | Low | Low | Low | Low | Low |
| Wubetu et al | Low | Low | Low | Low | Low | Low | Moderate | Moderate |
| Bilal et al | Low | Low | Low | Low | Low | Low | Low | Low |
| Geresu et al | Low | Low | Low | Low | Low | Low | Moderate | Moderate |
| Summoro et al | Low | Low | Low | Moderate | Moderate | Moderate | Low | High |
| Mamo and Alemu | Low | Low | Low | Low | Low | Low | Low | Low |
| Sisay et al. | Low | Low | Low | Low | Low | Low | Low | Low |
| Kasahun GG, et al. | Low | Moderate | Moderate | Low | Low | Low | Moderate | Moderate |
| Gashaw et al | Low | Low | Low | Low | Low | Low | Low | Low |
| Nigussie WD | Low | Low | Low | Low | Low | Low | Low | Low |
| Admassu Assen | Low | Low | Low | Low | Moderate | Moderate | Moderate | Moderate |
| Demeke B et al | Low | Low | Low | Low | Low | Low | Low | Low |
| Mosisa B et al. | Low | Low | Moderate | Moderate | Moderate | Low | Low | High |
| Mariam et al. | Low | Low | Low | Low | Low | Low | Moderate | Moderate |
| Gidebo et al | Low | Low | Low | Low | Low | Low | Moderate | Moderate |
| Desse et al | Low | Low | Low | Low | Low | Low | Low | Low |
| Gebramariam et al | Low | Low | Low | Low | Low | Moderate | Moderate | High |
| Hafte et al | Low | Low | Moderate | Low | Low | Low | Moderate | Moderate |
| Getahun et al | Low | Low | Low | Low | Low | Low | Low | Low |
